# Editorial: Severe acute respiratory syndrome coronavirus 2: Host-pathogen interactions and cellular signaling - vol II

**DOI:** 10.3389/fcimb.2023.1145235

**Published:** 2023-02-08

**Authors:** Vikas Sood, Clement Adebajo Meseko

**Affiliations:** ^1^ Department of Biochemistry, Jamia Hamdard, New Delhi, India; ^2^ Infectious and Transboundary Animal Diseases, National Veterinary Research Institute, Vom, Nigeria

**Keywords:** COVID-19, SARS-COV-2, coronaviruses, cellular signaling, host-pathogen interactions

The COVID-19 pandemic is cause by SARS-CoV-2, a betacoronavirus in the family Coronaviridae. Coronaviruses are enveloped, positive sense RNA viruses that are capable of infecting a diverse range of animals and humans. Coronaviruses infecting humans including 229E, NL63, OC43, and HKU1 could cause mild respiratory infection. Zoonotic and more pathogenic coronaviruses have emerged from animals in form of severe acute respiratory syndrome (SARS) and Middle East Respiratory Syndrome Coronavirus (MERS-CoV) in Saudi Arabia in 2002 and 2012 respectively. In 2019, SARS-CoV-2 evolved and spread across the globe causing a highly contagious infectious disease and responsible for the death of around 6 million people by the end of 2022. Thus SARS-CoV-2 has emerged as the most serious pandemic crisis since the 1918 pandemic (Agusi et al., 2020). Widespread and persistent circulation of SARS-CoV-2 contributes to host-pathogen interactions and cellular signaling. Consequently, and due to mutations, the virus has evolved rapidly and within three years, several variants have circulated across the globe. Some of those were designated “variants of concern” and predominate at different periods and regions with varying severity (https://www.who.int/news/item/26-11-2021-classification-of-omicron-(b.1.1.529)-sars-cov-2-variant-of-concern). Continuing emergence of new variants presents challenges for vaccine and antiviral drug development and studies in this special topic provide new information that will be useful for continuing efforts to blunt the impact of the virus. The success of vaccination and chemotherapy depends on a good understanding of the host and the pathogen interactions (Asha et al., 2019). The balance of this interaction determines the susceptibility of SARS-CoV-2 infection. Furthermore, the extent of the host-pathogen interaction affects the virulence or pathogenicity of the SARS-CoV-2 (Casadevall and Pirofski, 1999). In the course of invasion of the host cells, pathogens elicit both humoral and cellular immune responses and metabolic pathways that may contribute to eliminating invading pathogens (Forcados et al.). In addition, multiple cellular signaling pathways are elicited acting to reduce the severity of infection. Infection due to SARS-CoV-2, therefore, may be reduced in its clinical presentation, severity, and even mortality when a positive immune reaction is stimulated by pathogen-host interaction. It is imperative to delineate the mechanisms of viral entry, infections, and associated host immune response to efficiently manage SARS-CoV-2 infections as investigated in the collection of scientific articles in this special Research Topic. Insights into new aspects of coronavirus biology and pathogenesis will help identify novel cellular pathways that can be targeted for immunity.

In this special collection, Wang et al. reviewed angiotensin-converting enzyme 2 (ACE2), a receptor of SARS-CoV-2 as it mediates viral entry into cells, the spread, and initiation of infection. Soluble ACE2 (sACE2) has also been demonstrated to bind to SARS-CoV-2, and it functions in viral entry into cells and spread suggesting that sACE2 is increased in COVID-19 and correlates with disease severity. Regulating ACE2 shedding and understanding the underlying mechanism between sACE2 and the virus enlightens therapy for COVID-19. The characterization of the interaction between SARS-CoV-2 membrane protein (M) and proliferating cell nuclear antigen (PCNA) as a potential therapeutic target was also discussed by Zambalde et al.. The authors proposed that the transport of PCNA to the cytoplasm and its association with SARS-CoV-2 membrane protein could be a strategy used by the virus to manipulate cell functions and might be considered a target for COVID-19 therapy. For better understanding of downstream molecular signaling pathways induced by SARS-CoV-2 in the lungs of infected individuals, Baindara et al. described and examined the predictions of a model in which NOTCH may represent a central signaling axis in lung infection in COVID-19.

Plant-based drug discovery and targeted therapy is yet another approach that can be explored for discovering reliable, and efficient drugs against infections including SARS-CoV-2. The bioactive compounds of Ashwagandha [Withania somnifera (L.) Dunal] may be explored as herbal medicine for the management and treatment of patients infected by SARS-CoV-2 infection (Singh et al., 2022). In a study by Torrens-Mas et al., levels of circulating growth differentiation factor 15 (GDF15) and angiotensin-converting enzyme 2 (ACE2) in plasma of severity-stratified COVID-19 patients and uninfected control patients shows that circulating GDF15 and ACE2 stratify COVID-19 patients according to disease severity. Additionally, ACE2 missense SNPs constitute a risk factor linked to infection susceptibility.

Vaccination responses to the SARS-CoV-2 Omicron variant in the Chinese population were examined by Wu et al. The study was designed to determine whether vaccination could alter the disease course of the SARS-CoV-2 Omicron variant. The authors concluded that vaccination is associated with a shorter time to target cycle threshold value (TtCT) in patients with the SARS-CoV-2 Omicron variant. A brief research report provided insights from SARS-encoded non-coding RNAs. The authors performed in-silico analysis of SARS-CoV-2 genomes to identify SARS-CoV-2 encoded miRNAs. The study summary identified human and virus-encoded miRNAs that might regulate the pathogenesis of SARS coronaviruses and describe similar non-coding RNA sequences in SARS-CoV-2 that were shown to regulate SARS-induced lung pathology in mice (Periwal et al., 2022). In another study, cellular models to study SARS-CoV-2 and guide scientists venturing into studying the virus in the laboratories to choose the right cellular models were discussed (Pires De Souza et al., 2022). A study led by Scott et al. compared intracellular transcriptional response of NHBE cells to infection with SARS-CoV-2 Washington and New York Strains and suggest that some of the mechanisms associated with more severe disease from these viruses could include virus replication, metal ion usage, host translation shut off, host transcript stability, and immune inhibition.

Overall, this Research Topic highlighted the current research advancements made in the field of SARS-CoV2 Vol lI. The structural function and role of ACE receptors in the severity of infection was elucidated. The analysis of host-pathogen interaction is crucial for the development of effective antiviral strategies and vaccines for SARS-CoV-2 and the management of COVID-19.

## Author contributions

All authors listed have made a substantial, direct, and intellectual contribution to the work and approved it for publication.
